# Dyspepsia and Consumption

**Published:** 1896-02-29

**Authors:** Solomon C. Smith

**Affiliations:** Physician to the Westminster General Dispensary.


### Dyspepsia and Consumption.

By Solomon C. Smith, M.D., M.R.C.P., Physician to the Westminster General Dispensary.

### II.—THE FORMS OF DYSPEPSIA.

One of the most marked peculiarities seen in those whose indigestion ultimately terminates in consumption is a repugnance to fatty articles of diet; and although we may describe two quite different types of dyspepsia as preceding phthisis, in each of them the same dislike is found to occupy an important place.

Many people suffer from errors of digestion as a part of their very constitution, but the indigestion which is now in question is a malady coming on as a fresh condition. That a man always has had a feeble digestion may not mean much; but that a man who has had good health should begin to suffer with pain after his meals, acid risings, ending even in vomiting, that he should develop the red and pointed tongue indicative of irritable dyspepsia, should lose appetite and exhibit a loathing for fatty foods, and that along with all this he should betray a nervous despondency and anxiety about his every symptom which is quite foreign to his nature, is another affair; and if his occupation, surroundings, or family history are such as to suggest any predisposition to consumption, such symptoms are sufficient to make us very much concerned as to his future, however little may be discoverable on examining his lungs.

The same in regard to that more atonic form of dyspepsia which is especially apt to occur in young people. There are plenty of girls who are nibblers from their infancy, and never eat a square meal as their healthier sisters do. They are, perhaps, a feeble sort, but at least their requirements have become attuned to the powers of their digestions; but when after an illness or an accident, or, may be, after a mere nervous shock or a disappointment, a girl who has usually been accustomed to absorb and have the use of a fair amount of nutriment, falls into a state of debility, not merely affecting the muscular or nervous system, but touching her digestion, her very fount of energy, it is well to bear in mind that we, probably, have not to deal with a mere case of indigestion, but that the symptoms may be precursory of consumption.

What usually happens in such cases is that the patient loses appetite, strength, and flesh. The appetite is not only lessened, but is capricious, and generally there is an extreme dislike for fat, and sometimes also for saccharine and starchy forms of food. There is often discomfort and nausea after eating, with sourness and eructations, all of which are greatly increased if the taking of fat is insisted on, for the dislike to it is not a mere fancy, but is largely founded upon the fact of its disagreeing. Meanwhile the tongue is often furred, although its main peculiarity is its pallor and its flabbiness. The breath becomes offensive, the bowels confined, with pale offensive motions, there is great lassitude, the hands and feet being cold and often damp with perspiration. In girls also the catamenia are deficient and often absent altogether. It may only be after this sort of indigestion has continued for a long time that symptoms of consumption gradually intrude themselves.

When tuberculosis actually takes place new digestive troubles not uncommonly arise. In some cases no doubt the digestive disturbances of this phase of the disease are but the continuations of what has been going on before; but, even when there has been no precursory dyspepsia, a large majority of the cases now develop signs of indigestion, which often becomes a marked and troublesome complication at this stage of the disease. In regard to this it is also to be remarked that the stomach symptoms frequently so entirely predominate as to mask the gradually growing tuberculosis, a fact which has not uncommonly led to serious error in diagnosis.

Vomiting is often a marked symptom in this indigestion of early phthisis, and it is worth noting that while sometimes, especially in the early morning, it is brought on by the effort of coughing, in other cases the taking of food as definitely brings on the cough which persists until the stomach has emptied itself.

The first of these conditions does not matter very much, for after the morning vomiting is over a little sedative before each meal will suffice to hold the cough in check until the food has passed. The condition, however, in which the taking of food sets up uncontrollable cough, which, in turn, produces vomiting, is much more serious, as it very directly interferes with nutrition. A host of sedatives have been employed for the alleviation of this condition, of which the best probably are bismuth and alkaline effervescents, morphine and codeine, and alcohol, in the form of brandy and seltzer, before food. Merely washing the stomach by drinking warm water or the old-fashioned camomile tea a little before meals are due, sometimes makes it more tolerant of food. The fact is tolerably familiar to most of us that while the indigestions of the early stages of phthisis often get much better as the malady advances, yet, at a later stage, dyspepsia again asserts itself, and that this form of indigestion is apt to persist, notwithstanding every effort to relieve it, and to continue to the end. It seems, in fact, as if at a certain period of the disease the digestive organs failed in their functional activity and allowed the food to pass untouched.

Many have thought that this was the result of an irritation due to the repeated swallowing of the irritating discharges from the lungs, but, according to Saltau Fenwick, this terminal dyspepsia is the result of an interstitial gastro-enteritis, which arises from the absorption of toxic products from the cavities in the lungs. However this may be, it is the fact that the occurrence of anorexia and well-marked symptoms of dyspepsia in the later stages of the disease, after the stomach has appeared to throw off the gastric complications which may have marked its earlier steps, is a sign of serious moment, tending as it does to lessen the resisting powers of the patient at the very time when he requires them most.

